# Mechanisms and Functions of Chemerin in Cancer: Potential Roles in Therapeutic Intervention

**DOI:** 10.3389/fimmu.2018.02772

**Published:** 2018-11-30

**Authors:** Woo Jae Shin, Brian A. Zabel, Russell K. Pachynski

**Affiliations:** ^1^Division of Oncology, Washington University School of Medicine, St. Louis, MO, United States; ^2^Palo Alto Veterans Institute for Research (PAVIR), VA Palo Alto Health Care Systems (VAPAHCS), Palo Alto, CA, United States; ^3^The Andrew M. and Jane M. Bursky Center for Human Immunology & Immunotherapy Programs (CHiiPs), St. Louis, MO, United States

**Keywords:** chemerin, RARRES2, CMKLR1, CCRL2, GPR1, cytokine, chemoattractant, cancer

## Abstract

Chemerin [*RARRES2* [retinoic acid receptor responder 2], TIG2 [tazarotene induced gene 2 (TIG2)]] is a multifunctional cytokine initially described in skin cultures upon exposure to the synthetic retinoid tazarotene. Its secreted pro-form, prochemerin, is widely expressed, found systemically, and is readily converted into active chemerin by various proteases. Subsequent studies elucidated major roles of chemerin as both a leukocyte chemoattractant as well as an adipokine. Chemerin's main chemotactic receptor, the G-protein coupled receptor CMKLR1, is expressed on macrophages, dendritic, and NK cells. With respect to its role in immunology, chemerin mediates trafficking of these cells to sites of inflammation along its concentration gradient, and likely helps coordinate early responses, as it has been shown to have antimicrobial and angiogenic properties, as well. Recently, there has been mounting evidence that chemerin is an important factor in various cancers. As with its role in immune responses—where it can act as both a pro- and anti-inflammatory mediator—the potential functions or correlations chemerin has in or with cancer appears to be context dependent. Most studies, however, suggest a downregulation or loss of chemerin/*RARRES2* in malignancies compared to the normal tissue counterparts. Here, we perform a comprehensive review of the literature to date and summarize relevant findings in order to better define the roles of chemerin in the setting of the tumor microenvironment and tumor immune responses, with an ultimate focus on the potential for therapeutic intervention.

## Introduction

Chemerin [also known as retinoic acid receptor responder 2 (*RARRES2*) or tazarotene induced gene 2 (TIG2)] is a multifunctional, chemoattractant protein known for its roles in adipogenesis, angiogenesis, skin function, metabolic activity, and, recently, tumorigenesis. Initially secreted by the liver and white adipose tissue as prochemerin, the 163-amino acid precursor protein is readily cleaved by a specific set of serine proteases to become a chemotactically-active protein isoform of chemerin (1). Depending on the site of cleavage and subsequent interaction with its cognate heptahelical receptors, CMKLR1 (chemokine-like receptor 1), CCRL2 (C-C chemokine receptor-like 2), and GPR1 (G protein-coupled receptor 1), chemerin may exhibit varying degrees of bioactivity and elicit pro- or anti-inflammatory effects in different biological environments (1–3). In the literature, CMKLR1 is usually discussed as the primary receptor for chemerin interaction, while CCRL2 is described to participate in various functions by binding and presenting chemerin in a non-signaling manner to establish concentration gradients (4, 5). On the other hand, GPR1 is less-well characterized and found in the central nervous system (5) and reproductive organs (6, 7) and may play a role in metabolism (8).

Since its initial discovery in skin cultures upon stimulation with anti-psoriatic synthetic retinoid tazarotene, chemerin has been further described in a number of biological settings (9). In human endothelial cells, chemerin has been found to mediate angiogenesis via interactions with CMKLR1 (10), while, in the epidermis, chemerin has been shown to engage in significant antimicrobial activity (11). Moreover, chemerin has been linked with conditions such as obesity and diabetes, where it may modulate metabolism and adipocyte development (12, 13). Most recently, chemerin has been shown to mediate the chemoattraction of various immunocytes in the tumor microenvironment, while expression of chemerin's receptors, CMKLR1 and CCRL2, has been identified on a number of leukocyte subsets, namely, dendritic cell subsets, natural killer cells, and macrophages (1, 2, 14, 15). With recent findings that chemerin's receptors are also expressed on malignant tumor cells and that chemerin's expression is often altered in different cancer types, newer studies have focused on chemerin's novel roles in immune surveillance and tumor progression.

As expected, these recent studies have confirmed the notion that chemerin's functions in cancer are context driven. In some cancer types [e.g., glioblastoma, mesothelioma, neuroblastoma, squamous cell carcinoma of the esophagus, and squamous cell carcinoma of the oral tongue (SCCOT)], chemerin is upregulated (16–22). In most cancer types [e.g., acute myeloid leukemia (AML), adrenocortical carcinoma (ACC), breast cancer, Ewing sarcoma, hepatocellular carcinoma, melanoma, non-small cell lung cancer (NSCLC), prostate cancer, and squamous cell carcinoma of the skin] chemerin is downregulated, likely via hypermethylation of *RARRES2* (15, 19, 23–28) (Table 1). Adding to this complexity, the ultimate effects on tumor growth are also context-dependent; both tumor suppression and accelerated growth have been observed as a result of altered chemerin expression levels (Table 2). Thus, in this review, we attempt to catalog and analyze chemerin's specific functions in each tumor type, with a focus on identifying patterns in its mechanism of action and suggesting ways in which the chemerin system may be utilized or manipulated for clinical benefit.

**Table 1:** Alterations of chemerin expression profiles by cancer type.

**Cancer type:**	**Tumor expression of chemerin**	**Serum levels of chemerin**
Acute myeloid leukemia	↓	
Adrenocortical carcinoma	↓	↑
Breast cancer	↓	
Colorectal cancer		↑
Ewing sarcoma	↓	
Gastric cancer	↑	↑
Glioblastoma		
Hepatocellular carcinoma	↓	
Melanoma	↓	
Mesothelioma	↑	
Neuroblastoma	↑	
Non-small cell lung cancer	↓	↑
Prostate Cancer	↓	
Squamous cell carcinoma of the esophagus	↑	
Squamous cell carcinoma of the oral tongue	↑	↑
Squamous cell carcinoma of the skin	↓	

**Table 2:** Mechanisms of chemerin-mediated effects by cancer type.

**Cancer type**	**Model**	**Technique/method**	**Results**	**Chemerin-mediated effect**
Adrenocortical Carcinoma (28)	HEK293, H295R	BioCoat Matrigel invasion/migration assays	Transient overexpression of chemerin inhibited cell proliferation in HEK293, while inhibiting cellular invasion in KEK293, H295R	Inhibition of tumor cell proliferation and invasion
	H295R	Phosphokinase array +Western blot analysis	Phosphorylation and subsequent degradation of β-catenin and inhibition of p38 MAPK phosphorylation observed in stable, chemerin-overexpressing H295R ACC cell lines	Reduced β-cateinin and phosphorylated p38 MAPK levels
	H295R, HumanACC Tumor Samples	TCF/LEF Luciferase Reporter Assay + Immunohistochemistry	TCF/LEF reporter activity reduced in chemerin-overexpressing ACC cell lines compared to vector cell line; nuclear localized phosphorylated p38 signals detected in a majority of ACC tumor samples	Decreased activity of Wnt/ β-catenin and MAPK pathways
	Athymic Nude Mice, NSG Mice + H295R	Subcutaneous inoculation ofH295R in Athymic nude + NSG mice	Overexpression of chemerin significantly impaired tumor growth and resulted in lower tumor weight in both mouse models	Suppression of tumor growth
Gastric Cancer (29)	AGS, MKN28	Matrigel-coated Transwell assay	Increased invasion of AGS and MKN28 cells through Matrigel-coated Transwells at extremely low concentrations (0.0 I ng/ml)	Enhanced tumor invasion
	AGS, MKN28	Real time-PCR	Induction of mRNA expression of pro-invasive genes, IL-6, VEGF, via chemerin in AGS and MKN28, and also MMP-7 in MKN28	Increased expression of IL-6, VEGF, MMP-7
	AGS, MKN28	Western blot analysis	Increased phosphorylation of p38, ERKl/2 via chemerin in AGS, MKN28	Increased activity of MAPK pathways (MEK-ERK, MKK3/6-p38)
Glioblastoma (17)	U87MG	Calcium mobilization assay	Chemerin stimulation induced a transient, dose dependent increase of intracellular calcium in U87 MG cells	Induction of intracellular calcium
Hepatocellular Carcinoma	C57BL/6 Mice +Hepal-6	Implantation of HCC hepal-6 in C57BL/6 mice + subcutaneous model	WT mice with chemerin-expressing Hepa 1-6 tumors had a lower mortality rate and liver tumor growth/weight compared to Rarres2^−/−^ mice with Hepa1-6 tumors	Inhibition of tumor growth
	C57BL/6 Mice +Hepa1-6	Intravenous injection of hepal-6 in C57BL/6 mice	WT mice with chemerin-expressing Hepa1-6 tumors had less metastatic nodules compared to Rarres2^−/−^- mice with Hepal-6 tumors	Reduced lung metastasis
	C57BL/6 Mice +Hepa1−6	Flow cytometry	Rarres2^−/−^ mice with Hepa1-6 tumors showed heightened proportions of MDSCs and TAMs and decreased levels ofCD4/CD8 T cells compared to WT mice with chemerin-expressing Hepal-6	Reduced induction of MDSCs and TAMs and increased accumulation of tumor infiltrating CD4/CD8 T cells
	C57BL/6 Mice +Hepal-6	Quantitative reverse transcriptase-PCR	Chemerin-expressing Hepal-6 tumor cells exhibited significantly decreased expression ofGM-CSF, IL-6	Reduced expression of GM-CSF + IL-6
Li et al. (30)	7404, HepG2	Boyden chamber + Transwell invasion assay	Overexpression of chemerin resulted in reduced migratory ability and invasiveness of 7404 cells, whereas chemerin knockdown enhanced these properties in 7404/che Hand Hep G2 cells	Reduced migratory ability and invasiveness
	BALB/c Mice +PVTT-1	Left ventricular + intrahepatic injection model	Prolonged survival and reduced/delayed appearance of metastatic foci was observed in mice injected with PVTT−1 che cells (chemerin overexpressing), compared to mice injected with PVTT−1 con cells in both injection models (Resuts were replicated by regularly injecting chemerin in mice with PVTT-1 tumors)	Inhibited metastasis and prolonged survival times (Reduced weight loss)
	HCCTMA	Immunohistochemistry	High levels of chemerin were correlated with low levels of p-Akt (Ser473) and high levels of PTEN in HCC TMA	Reduced Akt/MMP 1 as a result of positive regulation of PTEN via chemerin
	7404, PVTT-1, Hep3B, HepG2	Western blot analysis	In chemerin-overexpressing HCC cells (7404, Hep3B, and PVTT-1), reduced p-Akt(Ser473) levels were observed, while elevated p-Akt(Ser473) levels were observed in chemerin knockdown HCC cells (7404/che H, HepG2)	Downregulation ofp-Akt (Ser473)
Melanoma (15)	C57BL/6 Mice +Bl6	Flow cytometry	The average frequency of NK and T cells were increased in chemerin- expressing tumors	Enhanced infiltration of antitumor leukocytes
	C57BL/6 Mice C57BL/6 Mice	Subcutaneous inoculation ofB16 murine melanoma in C57BL/6 Mice	Chemerin-expressing melanomas exhibited delayed growth compared to control transfectants (measured by tumor size)	Impaired tumor growth
Neuroblastoma (21)	SK-N-SH	Confocal laser scanning microscopy	Fast, transient increase in intracellular calcium observed in SK-N-SH cells following stimulation with lOnM chemerin	Induction of intracellular calcium
	SK-N-AS, SK-N-BE(2)	Western blot analysis	Increased, dose dependent phosphorylation of Akt, MEKl/2, ERKl/2 in cell lines observed after stimulation via chemerin	Increased activity of MAPK and Akt pathways
	SK-N-AS, SK-N-BE(2)	Real-time zymography	Increased synthesis ofMMP-2 in cell types after 6, 12, 24, 48 h stimulation with chemerin	Increased expression of MMP-2
Non-Small Cell Lung Cancer (31)	LLCI	Flow cytometry	Splenocytes cultured with prochemerin-expressing Lewis Lung Cancer cells (LLC) exhibited decreased levels ofTNF-a, IL-12 p40 and slightly increased levels of IFN-*y*	Decreased expression of inflammatory cytokines, TNF-a, IL-12 p40
	C57BL/6 Mice +LLCI	Subcutaneous inoculation of Prochemerin-LLCI into C57BL/6 mice	C57BL/6 mice with prochemerin-expressing LLC tumors exhibited a lower incidence of tumor formation, but not a decrease in tumor growth rate	Prochemerin induced inhibition of tumor formation
Squamous Cell Carcinoma of the Esophagus (20)	OE21	Matrigel-coated boyden chamber invasion assay	Enhanced invasion of OE21 cells in Matrigel-coated Boyden chambers upon stimulation with chemerin (effects abrogated by CCX832, a CMKLRI inhibitor)	Enhanced tumor invasion
	OE21	Western blot analysis	Increased expression of MMP-1, MMP-2, MMP-3 in OE21 cells stimulated by myofibroblast-secreted chemerin (effect mediated by protein kinase C; MMP responses inhibited by Ro31822, a PKC inhibitor)	Increased expression of MMP-l, MMP-2,MMP-3
	OE21	Enzyme activity assay	Increased activity ofMMP-1, MMP-2, MMP-3 (measured in fluorescence intensity) in OE21 cell media, induced by chemerin	Increased activity of MMP-1, MMP-2, MMP-3
Squamous Cell Carcinoma of the Skin (19)	SCL-1, SCC-12B2, SCC-13, A431	Transwell Chamber Migration assay	Significant increase in cell migration (cells/HPF) induced by I 0, 20, 40 nM of recombinant human chemerin (SCL-1), 20,40 nM (SCC-12B2), 10, 20, 40 nM (SCC-13), 20 nM (A431)	Enhanced tumor migration
	SCL-1	GPCR Cignal Finder array	Enhanced MAPK activity of JNK and ERK signaling pathways after I hour of stimulation via recombinant human chemerin	Increased activity of MAPK pathways (JNK, ERK)
	SCL-1	Western blot analysis	Increased phosphorylation of ATF2, c-Jun, SEK1, ERKI/2 observed in chemerin treated SCL-1 cells compared to untreated SCL-1 cells	Increased activity of MAPK pathways (JNK, ERK)

## Cancers

### Acute myeloid leukemia

Acute myeloid leukemia (AML) is the most prevalent form of acute leukemia affecting adults, with a particularly high rate of incidence (12.2 cases per 100,000 people) for those above the age of 65 (32–34). Though recent advances in AML treatments have significantly improved prognosis for younger patients, those who are older have little chance of survival (35). In fact, a majority of AML patients over the age of 65 die within a year of diagnosis, underlying an urgent need for improved methods of detection that may enable earlier life-preserving treatment (36).

In light of this need, chemerin has recently been identified as a potential biomarker for diagnosis of AML. A study in 2017 revealed that chemerin was downregulated in the bone marrow mononuclear cells of AML patients compared to that of healthy controls, with ROC curve analysis showing a test specificity of 79% (true positive) and sensitivity of 54% (true negative) for chemerin expression as a marker for AML diagnosis. The same group performed a cohort study of 149 patients, 32 of whom had high chemerin expression and 117 of whom had low expression, with no significant variability in certain gene mutations, white blood cell count, platelets, and hemoglobin, and found that patients with low chemerin expression correlated with poorer overall survival. Moreover, multivariate analysis on parameters such as age, various gene mutations, chemerin expression, karyotypic classifications, and white blood cell count verified that chemerin was independently able to prognosticate AML patients, while univariate analysis of chemerin expression levels showed that high chemerin expression was associated with positive prognosis (22). In terms of chemerin receptor expression levels (independent of associations between chemerin expression and clinical outcome), a different group showed that CCRL2 was overexpressed in AML, identifying the non-classically signaling chemerin receptor as a potential therapeutic target, along with other GPCRs that were also overexpressed (37).

### Adrenocortical carcinoma

Adrenocortical carcinoma (ACC) is an extremely rare and aggressive tumor that is associated with poor prognosis in patients. The incidence rate for ACC is 0.7–2.0 cases per million people per year, and for patients that undergo first-line treatment (surgical resection), the 5-year median survival rate is 38.6%. For patients ineligible or unwilling to undergo adrenalectomy, there is little chance of remission (38–40). In fact, the potential risk of ACC is considered the standard for adrenalectomy in patients with adrenal incidentalomas (38).

Recent research suggests that serum chemerin levels have prognostic value in ACC and that manipulating chemerin levels in ACC tumors may prove efficacious in ACC patients. Chemerin's role as a chemoattractant, in recruiting immune cells to sites of inflammation, has already been well documented; for instance, chemerin has been shown to suppress neoplasia by eliciting natural killer cells to the tumor site in melanoma (15). In line with those findings, a study by Chittenden et al. (28) featuring a group of 20 ACC patients, 53 benign tumor patients, and 21 healthy individuals reported that serum chemerin levels were elevated in ACC patients as compared to those with benign adrenocortical tumors, and the difference further increased when tested against healthy controls. They also discovered that higher serum chemerin levels were strongly associated with better overall survival. Though seemingly paradoxical, it was proposed that two important, proven factors may explicate this phenomenon. First, a mouse xenograft model showed that increased serum chemerin levels were not a result of secretions from the tumor itself but from the host environment (28). Second, as mentioned previously, chemerin has been shown to be an effective recruiter of immune cells to sites of inflammation (1, 14, 15, 41). Thus, it was suggested that ACC may downregulate chemerin as a method of immune evasion and that the host environment may increase secretion of chemerin in serum as a counteractive response (28).

Interestingly, in addition to the potential for immune-dependent methods of tumor suppression, a new study has found evidence that chemerin may directly alter malignant cells in an immune-independent manner. A study by Liu-Chittenden et al. (42) found that the RARRES2 gene was transcriptionally downregulated in ACC, in line with similar findings in other cancer types. Specifically, the silenced RARRES2 gene in ACC tumors was characterized by hypermethylation at five CpG sites. This was confirmed in three human ACC cell lines and HEK293 cells via treatment with a DNA methyltransferase inhibitor, which showed that such treatment reversed the effects of methylation at all CpG sites in a dose dependent manner (42, 43). [As an aside, in Ewing sarcoma, a rare, malignant tumor that grows inside the bones and in nearby soft tissues, RARRES2 showed a high rate of methylation and was one of only eight genes to have a frequency of silencing >20% (25). Thus, hypermethylation of RARRES2 is likely a common method of gene silencing in tumors where chemerin is downregulated].

When ACC cell lines were transfected to express chemerin, significant inhibition of tumor growth was observed in a dose dependent manner, as the cell lines that had higher expression of chemerin correlated to a more compelling reduction in tumorigenesis (42). Supporting these results, exogenous addition of recombinant chemerin *in vitro* showed no meaningful effect in affecting cell proliferation among various ACC cell lines, implying that the mechanism of growth inhibition was not mediated by the binding of chemerin to its receptors, such as CMKLR1 (42). In fact, chemerin's function in reducing ACC tumor growth was found to be mediated by two different, immune-independent mechanisms. First, chemerin overexpression could induce β-catenin phosphorylation, and thus, proteasome mediated degradation. In phosphokinase arrays of a chemerin overexpressing ACC cell line, significant reduction in total β-catenin levels was observed, whereas treatment with a proteasome inhibitor prevented proteasome mediated degradation, allowing for detection of elevated phosphorylated β-catenin levels (42). The phosphorylated sites were identified as Ser33, Ser37, and Thr41. Consequently, a decrease in Wnt/β-catenin pathway activity was observed and confirmed via a TCF/LEF luciferase reporter assay (42). Second, chemerin overexpression could inhibit p38 MAPK phosphorylation. The phosphokinase array, which showed a decrease in total β-catenin levels, also showed a reduction in phospho-p38 MAPK levels (42). *In vivo* xenograft studies in athymic nude mice and NSG mice, both immunodeficient mouse models, confirmed that chemerin overexpression resulted in lower β-catenin and phosphorylated p38 MAPK levels (42). Together, these results indicated that chemerin could promote tumor suppression through immune-independent pathways, both *in vitro* and *in vivo*.

These findings may have positive implications for ACC patients and other cancer patients alike. β-catenin (CTNNB1), a proto-oncogene, is frequently mutated in ACC, resulting in constitutive activation of the Wnt/β-catenin pathway (44). The occurrence of activating mutations of CTNNB1 is generally known to be a significant pathway for ACC tumorigenesis. Indeed, a majority of adrenocortical tumors exhibit activation of the Wnt/β-catenin pathway, which is correlated with poor outcomes in ACC patients (45, 46). Aberrant activation of the Wnt/β-catenin pathway is common in many other cancer types, such as breast cancer, lung cancer, hepatocellular carcinoma, and squamous cell carcinoma (47). Additionally, elevated phospho-p38 MAPK levels are found in a majority of adrenocortical tumors (42). In other cancer types such as lung cancer and colon cancer, abnormal activation of p38 signals are detected (48, 49). As such, it is reasonable to suspect that chemerin may have similar, tumor-suppressive effects in other cancer types.

Finally, chemerin may also serve as a prognostic marker for ACC. Among patients, tissue chemerin levels did not correspond to prognosis, though chemerin expression was downregulated in ACC tissue samples (28). However, serum chemerin levels did correspond to prognosis. A clinical survey showed that serum chemerin levels were significantly elevated in ACC patients in comparison to patients with benign tumors and even more so in comparison to healthy controls. Survival analysis of median serum chemerin levels in ACC patients determined that higher serum chemerin levels correlated with longer survival. Moreover, prognosis for patients with recurrent ACC could be stratified according to varying serum chemerin levels, further verifying its value as a marker for ACC prognosis (28).

### Breast cancer

Breast cancer is the most common malignancy in women, accounting for a quarter of all cancer cases in females (50). It is estimated that one in every eight women will develop breast cancer at some point in their lives (51). In this context, chemerin has not been deeply investigated; though, there are some preliminary pieces of evidence showing that chemerin is significantly downregulated in breast adenocarcinomas, one of the most common forms of breast cancer (15). Additionally, studies have detected CMKLR1 in breast tissue (52). Taken together, these facts raise the suspicion that chemerin may exhibit antitumor effects in breast cancer.

However, pro-tumor effects are also possible. Given the increased risk of breast cancer due to post-menopausal obesity and chemerin's known associations with obesity and obesity related parameters, such as blood pressure and BMI, some suspect that chemerin may be correlated to higher risk of breast cancer, albeit indirectly (51, 53). Moreover, it has been experimentally shown that some adipose-derived angiogenic factors may promote breast cancer growth, though this remains to be tested in the context of chemerin (54, 55). It has also been speculated that chemerin may play a role in instigating metastasis through its angiogenic functions (51). For prognosis, it is unclear whether chemerin can be used as a biomarker for breast cancer. A cross sectional study involving 117 breast cancer patients by Akin et al. (51) showed that serum chemerin levels could not be used in staging of breast cancer, as there were no differences in serum chemerin levels of patients with metastatic and non-metastatic cancer.

### Colorectal cancer

Colorectal cancer has the third highest incidence rate and fourth highest mortality rate of all cancers types, with 1.4 million new cases and 700,000 related deaths per year (56). Current treatment methods range from chemotherapy (e.g., single-agent 5-fluorouracil, oxaliplatin, and/or irinotecan) to other regimens consisting of newer, targeted substances, such as anti-vascular endothelial growth factor-A antibodies or anti-epidermal growth factor receptor antibodies (57).

Among the early diagnostic tools that have become available in recent years, serum chemerin levels have been identified as an effective biomarker for colorectal cancer. A study in 2018 compared 32 colorectal cancer patients with sex, BMI, and age matched healthy volunteers and reported that chemerin was significantly upregulated in serum of colorectal cancer patients, increasing with higher tumor stage progression (58). The area under the receiver operating characteristic (ROC) curve for serum chemerin levels was 1, with a sensitivity and specificity of 100% (58). The results clearly indicated that serum chemerin could be an effective biomarker for colorectal cancer and stage progression.

Additionally, a pilot study of 110 colorectal cancer survivors in Korea showed that there was an inverse relationship between serum chemerin levels and colorectal cancer-related quality of life, defined in terms of Functional Assessment of Cancer Therapy (FACT)-General, -Colorectal, and -Fatigue scores (59). Given that chemerin has been shown to have antimicrobial properties, it was proposed that chemerin may play a role in modulating gut microbial activity, which may play a role in the various bowel symptoms affecting colorectal cancer patients (11, 60). Supporting this hypothesis, colorectal cancer patients who have undergone resection surgery have consistently reported altered gut microbial activity, which has been linked to numerous bowel symptoms in these patients (61–63). Interestingly, the gut microbiome is somewhat altered in chemerin KO mice compared to WT controls, with an increase in Desulfovibrionaceae (64). Increases in this pathobiont have been reported as a component of IBD-associated gut dysbiosis (65).

### Gastric cancer

Gastric cancer is the fourth most common cancer and the second most common cause of cancer-related death in the world (66). Treatment of gastric cancer generally consists of a combination of surgery and chemotherapy (66). Though current clinical parameters for diagnosis are incapable of accurately predicting patient outcomes, recent evidence has revealed chemerin's prognostic value in gastric cancer, as well as its functions in tumor growth (67).

A 2014 study comparing 196 gastric cancer patients with 196 age- and sex-matched healthy controls showed that preoperative plasma chemerin levels were significantly elevated in cancer patients (67). Multivariate analysis of clinical parameters currently used for diagnosis and serum chemerin levels showed that serum chemerin could be used as an independent predictor of 5-year mortality, 5-year adverse event, overall survival, and 5-year disease free survival, though further research is needed to adjust for the possibility of postoperative alterations (67).

In terms of functional roles, studies have shown that chemerin exhibits pro-tumor effects in gastric cancer, confirming the notion that chemerin's pro/anti-tumor functions are context dependent. Indeed, elevated serum chemerin levels were observed in gastric cancer patients and were shown to be correlated with increased tumor invasiveness (29). Specifically, chemerin contributed to tumor growth by inducing phosphorylation of p38 and ERK 1/2 MAPKs and upregulating IL-6, MMP-7, and VEGF (29). Chemerin treatment did not alter the *in vitro* proliferation of two human gastric cancer cell lines, AGS and MKN28. However, chemerin increased the invasiveness of both cell types through Matrigel-coated transwells with only miniscule concentrations of chemerin. In fact, the number of cells invading through the membrane increased fivefold or more for AGS and MKN28 cells exposed to chemerin. Moreover, chemerin increased mRNA expression of VEGF and IL-6 in AGS and MKN28 cells, while also increasing expression of MMP-7 exclusively in MKN28 (29). VEGF, IL-6, and MMP-7 have all been associated with enhanced tumor invasiveness in gastric cancer (68–70), while high expression of VEGF and IL-6 have been shown to stimulate metastasis of malignant cells and indicate poor clinical outcomes in gastric cancer patients, suggesting a potential impact of chemerin in this setting (69–72).

In addition to increased expression of various pro-invasion genes, stimulation of AGS and MKN28 via chemerin resulted in elevated phosphorylation of p38 and ERK1/2 in both cancer lines, suggesting that chemerin could activate MKK3/6-p38 and MEK-ERK MAPK signaling cascades (29). Importantly, these are known mechanisms by which tumor cells modulate growth and invasion in gastric cancer (73–76). Elevation of phosphorylated ERK 1/2 levels have been associated with poor survival in patients, and p38 activation has been found to incite peritoneal dissemination in gastric cancer (75, 76). Given that these effects were abrogated by treatment with inhibitors of MEK-ERK signaling, it was clear that the MEK-ERK MAPK pathway was primarily associated with increased gastric cancer invasiveness (29). Together, the results of the studies indicated that chemerin may engender pro-tumor effects in gastric cancer, while also serving as a marker for patient prognosis.

### Glioblastoma

Glioblastoma is the most malignant form of brain tumors affecting adults (77). Recent advances in glioblastoma research have made new treatment methods available to patients, such as tumor-treating fields and immunotherapy. However, the classical approach to treating glioblastoma remains a multipronged effort, using a combination of surgical resection, radiation, and chemotherapy (77). The incidence rate of glioblastoma hovers around 3.19 cases per 100,000 people in the United States and is very uncommon in children (78).

Though chemerin's role in glioblastoma is still unclear, studies have reported an altered chemerin profile in glioblastoma cases (17). First, one study found that chem158 K, a bioactive isoform of chemerin, was elevated in the cerebrospinal fluid of patients with malignant glioblastoma (79). A later study found that chemerin mRNA expression was significantly increased in grade 3 and 4 glioma, equivalent to malignant glioblastoma, compared to epilepsy and grade 2 glioma (17). In contrast, CMKLR1 and CCRL2 mRNA expression was unaltered in those cases. To further test whether chemerin could function as a signaling molecule, *in vitro* experiments were conducted using U-87 MG cells, a human glioblastoma cell line. Experimental results determined that the addition of chem157S, another bioactive isoform of chemerin, to U-87 MG cells resulted in a transient, dose-dependent increase of intracellular calcium, indicating that chemerin could instigate intracellular signaling in U-87 MG cells (17). Whether these results translate to experiments *in vivo* remains to be seen.

### Hepatocellular carcinoma

Hepatocellular carcinoma (HCC) is the fifth most prevalent cancer in the world, with more than 500,000 new cases being diagnosed each year (80). It is mainly caused by chronic liver damage due to hepatitis B or hepatitis C and is associated with risk factors such as obesity and diabetes (80). Currently, a variety of treatment options are available to patients, including liver transplantation, curative resection, radiotherapy, radio/chemo-embolization, and systemic therapies (81).

In recent years, chemerin has been identified as a potential therapeutic agent for HCC. A study comparing chemerin expression levels in 124 HCC patient tumors and matched, normal tissues showed that chemerin was significantly downregulated in 72 of the patients' liver tissues and that tissue chemerin expression correlated with tumor size, grade, and infiltration of dendritic cells and natural killer cells. Additionally, chemerin was identified as an independent factor for prognosis via multivariate analysis, with lower chemerin expression corresponding to poorer overall survival (23).

A later study by Lin et al. (82) further reported that chemerin could inhibit HCC tumor growth. In an orthotopic mouse model of HCC, chemerin knockout mice showed aggressive tumor growth and metastasis. In contrast, overexpression of chemerin in mice resulted in delayed tumor growth, suggesting that chemerin may hamper tumor progression. Specifically, the inhibitory effect was mediated by suppressing pro-tumor inflammatory cytokines. When Hepa1-6 cells were treated with chemerin *in vitro*, cell survival and proliferation was unaffected. Moreover, chemerin knockout mice with accelerated tumor growth exhibited increased expression of granulocyte-macrophage colony-stimulating factor (GM-CSF) and IL-6 and accumulation of myeloid-derived suppressor cells (MDSCs) and tumor-associated macrophages (TAMs). Neutralization of GM-CSF and IL-6 reversed the effects in the chemerin knockout mice, showing that these two cytokines were mainly responsible for the accelerated growth. Combined with the fact that serum chemerin levels were inversely correlated to GM-CSF and IL-6 expression levels in patients with HCC, the study suggested that chemerin may have a negative regulatory role for these two cytokines (82).

Further verifying these claims, chemerin was shown to inhibit nuclear factor-κB activation, an important factor for GM-CSF and IL-6 expression (83, 84). The data consistently showed that chemerin targeted tumor cells and tumor-associated endothelial cells, the major source of GM-CSF and IL-6, via interaction with CMKLR1 and CCRL2, reducing expression of inflammatory cytokines and inhibiting NF-κB activation (82). Downregulation of CMKLR1 and CCRL2 via siRNA in Hepa1-6 cells reestablished GM-CSF and IL-6 expression, indicating that decreased expression of the cytokines was mediated by chemerin-CMKLR1/CCRL2 interactions. The observed consequences of reduced GM-CSF and IL-6 expression were impaired MDSC accumulation, reestablishment of anti-tumor IFN-γ^+^ T-cell activity, and hampered tumor angiogenesis (82).

The inhibitory function of chemerin in HCC was mediated by both T-cell dependent and independent mechanisms. Because chemerin showed less of an inhibitory effect in Rag1 knockout and CD8-T-cell depleted mice, it was determined that chemerin's function in HCC was not completely based on T-cell anti-tumor activity (82). It was postulated that chemerin's T-cell independent mechanism of inhibition was mediated by reduced accumulation of MDSCs, which are a major source of pro-angiogenic factors Bv8 and MMP-9. Supporting this hypothesis, overexpression of chemerin in mice resulted in a notable decrease in Bv8 and MMP-9 expression and, ultimately, tumor angiogenesis (82).

A recently published study showed a novel interaction of chemerin and the tumor suppressor PTEN in a mouse model of HCC (30). *In vitro*, overexpression of chemerin resulted in reduced migratory ability and invasiveness of human HCC line 7404. Chemerin knockdown, in turn, resulted in increased migratory ability and invasiveness. *In vivo*, overexpression of chemerin inhibited intra- and extrahepatic metastases of HCC cells in nude mice, lengthening survival times of HCC inoculated mice. Nude mice with chemerin overexpressing tumors showed markedly less distant metastases and delayed appearance of metastatic foci using control or chemerin-expressing PVTT-1 HCC cells. Consistent observations were made in an intrahepatic injection model. Moreover, regular intraperitoneal injections or intrahepatical injections of chemerin in mice with PVTT-1 tumors replicated these results, with the additional benefit of reduced weight loss, showing that chemerin could potentially be therapeutically administered to suppress HCC metastases.

Underlying these tumor suppressive effects, the mechanism of reduced HCC cell migration and invasion via chemerin was shown to be mediated by negative regulation of p-Akt (Ser473) by PTEN. P-Akt and MMP-1 levels, as well as ubiquitination of PTEN, were reduced in chemerin-overexpressing HCC cell lines and elevated in chemerin-knockdown HCC cell lines. PTEN levels were upregulated in chemerin-overexpressing HCC cell lines and downregulated in chemerin-knockdown HCC cell lines. Immunohistochemistry of HCC tissues showed that upregulation of chemerin correlated with low p-Akt and high PTEN levels. Supporting these findings *in vivo*, data from their tumor models showed that overexpression of chemerin was associated with reduced tumor MMP-1 and p-Akt levels, elevated PTEN levels, and reduced metastases. Taken in aggregate, these studies provided strong support for therapeutic applications of chemerin in HCC.

### Melanoma

Melanoma has one of the fastest growing incidence rates in the world, which in the US has risen from 8.2–9.4 cases per 100,000 people in 1975 to 24.2–35.4 cases per 100,000 people in 2010 (85). Unlike many malignancies, melanoma affects a diverse range of age groups, with a median age of diagnosis of 57 years (86). It is also widely recognized as the most aggressive form of skin cancer, accounting for the majority of skin cancer-related deaths vs. a fraction of total cases (86). Significantly, recent research has elucidated chemerin's ability to induce tumor suppression in melanoma, where chemerin was found to be downregulated (15).

Our group previously identified the mechanism of tumor suppression via chemerin in melanoma, showing that tumor expression of chemerin led to inhibited tumor growth *in vivo*. When chemerin-expressing B16F0 (hereafter, B16) cells were implanted into mice, the mouse melanoma showed significant delay in growth compared to control B16 cell lines. Importantly, the RARRES2-transfected B16 cells expressed bioactive chemerin, confirmed via chemotaxis, and both the transfected line and control B16 lines lacked expression of CMKLR1. When tested *in vitro*, chemerin treatment failed to alter B16 cell proliferation, suggesting that chemerin's inhibitory effects in melanoma was mediated by host immune responses (15). It was also noted that chemerin expressing tumors exhibited enhanced infiltration by NK and T cells; ratios of NK and T cells to MDSCs and/or plasmacytoid dendritic cells (pDC) were also increased (15, 87, 88). Given that certain subsets of pDCs and MDSCs have been reported to be tolerogenic, suppressing the body's antitumor immune responses, it was noted that increased ratios of antitumor immune cells to tolerogenic ones would translate to a more favorable environment for tumor suppression (89, 90). Moreover, chemerin's tumor suppressive effects in melanoma were mediated by NK cells, as their depletion abrogated the antitumor effects, while a lack of T cells and B cells did not alter the tumor growth-inhibition phenotype (15). Further experiments showed that host expression of CMKLR1 was necessary for inhibited tumor growth, since chemerin-expressing mouse melanoma grew faster than control B16 tumors in CMKLR1-negative mice. Finally, local administration of chemerin suppressed tumor growth *in vivo* (15).

Thus, in melanoma, chemerin was shown to inhibit tumor growth by eliciting antitumor responses and altering the tumor microenvironment in favor of growth inhibition. Whether by tumoral expression or local administration, these tumor-suppressive effects could be observed *in vivo* (15). Additionally, high expression of chemerin was shown to be associated with better outcomes for patients in two clinical studies, demonstrating chemerin's potential for therapeutic intervention in melanoma (15).

### Mesothelioma

Mesothelioma is a tumor that grows in the linings of various organs, such as the lungs or the heart. Tumorigenesis is instigated by exposure to specific, carcinogenic mineral fibers, namely asbestos (63). Due to the commercial use of such materials, the incidence of mesothelioma has increased over the years, from near non-existence to several thousand cases per year (63). Though treatments are available, such as chemotherapy with cisplatin and pemetrexed, prognosis is still dismal and diagnosis very difficult, as it may take several decades for symptoms to appear (91). Regarding chemerin's role in mesothelioma, literature on the topic is scarce, though it was one of the first identified cancer types where RARRES2 expression was altered (significantly increased, compared to matched normal tissue) (16). It remains unclear if chemerin contributes in any way to mesothelioma progression.

### Neuroblastoma

Neuroblastoma is a pediatric cancer affecting the sympathetic nervous system. With < 50% probability of cure for high-risk cases, the prognosis for children with advanced stage neuroblastoma is bleak (92). For those with high-risk neuroblastoma, treatment is intensive, consisting of several modalities, such as chemotherapy, surgery, immunotherapy, stem cell rescue, and differentiation therapy (93).

A recent study by Tümmler et al. (21) showed that expression of chemerin receptors could successfully prognosticate neuroblastoma. Based on data from public datasets, the study found that high expression of GPR1 and CMKLR1 was associated with low survival rates. Furthermore, expression of CMKLR1 and CCRL2 was found to be upregulated in neuroblastoma cohorts in comparison to benign counterparts, while increased CCRL2 expression was correlated to poor prognosis. Increased chemerin expression was also found in neuroblastoma cohorts in comparison to neural crest, though no differences in expression levels were found between neuroblastoma and benign neurofibroma. Overall, CMKLR1 and GPR1 expression was shown to be a potentially viable indicator of prognosis in neuroblastoma patients (21).

According to the same study, inhibition of the chemerin/CMKLR1 axis exhibited antitumor effects *in vitro* and *in vivo*, suggesting that the chemerin/CMKLR1 axis could be a potential therapeutic target for neuroblastoma. *In vitro*, expression of CMKLR1, chemerin mRNA/protein, and GPR1 was verified in neuroblastoma cell lines (as well as primary tumors) via real time-PCR and western blot analysis (21). Exposure to inflammatory cytokines TNF-α, IL-1β, and serum resulted in increased secretion of chemerin by neuroblastoma cells. Moreover, the mechanistic effects of added chemerin in neuroblastoma cell lines was the induction of intracellular calcium, activation of MAPK and Akt signaling, and synthesis of MMP-2. Specifically, in a human neuroblastoma cell line (SK-N-SH), chemerin induced a rapid increase in intracellular calcium. In SK-N-AS and SK-N-BE(2) cells, a dose-dependent increase in MEK 1/2, Akt, and ERK 1/2 phosphorylation was observed upon addition of chemerin, indicating the activation of Akt and MAPK pathways. In the same cell lines, a dose-dependent increase in MMP-2 synthesis was shown via real-time zymography, after cells had been stimulated via chemerin for various timepoints. Significantly, inhibition of CMKLR1 on four neuroblastoma cell lines via α-NETA, a recently described CMKLR1 inhibitor (94), dose-dependently reduced cell viability and clonogenicity (21). Similar results were achieved *in vivo*. In a SK-N-AS xenograft model, mice that were pretreated with α-NETA, before tumors reached a specified volume, showed longer survival and delayed tumor growth compared to control mice (21). Thus, the results of both *in vitro* and *in vivo* experiments indicated that targeting chemerin/CMKLR1 could potentially elicit antitumor effects in clinical settings.

### Non-small cell lung cancer

Lung cancer is the leading cause of cancer-related deaths in the world, having a 5-year survival rate of < 15% and resulting in ~1.4 million deaths per year (66, 95). This is despite the fact that various chemotherapy-based treatment methods are available to patients. For non-small cell lung cancer (NSCLC), which accounts for the majority (~85%) of lung cancer cases, multiple studies indicate that chemerin may be of great diagnostic and prognostic value (24, 27, 96). Two independent studies, one in 2011 and another in 2016, reported that chemerin was downregulated in NSCLC (24, 27). Zhao et al. (24) compared 108 NSCLC tumor samples with corresponding disease-free tissues and found that 56 of the NSCLC patient tumors showed a lower chemerin expression profile. They also found a positive correlation between chemerin expression levels and infiltration of NK cells into tumor, as revealed by CD56 IHC staining of NSCLC patient tissue samples. Moreover, multivariate analysis of various parameters, such as age, smoking history, tumor size, and differentiation grade, and univariate analysis, together, revealed that lower levels of chemerin expression in tissue were significantly associated with tumor-node metastasis stage, degree of differentiation, and poorer survival rates (24). In line with these findings, a 2016 study by Cai et al. (27) comparing 20 NSCLC tumor samples with their corresponding non-tumor tissues found that 16 of the 20 tumor samples exhibited a significant downregulation of chemerin. Chemerin mRNA expression was also tested in 26 NSCLC tumor samples along with their healthy tissue counterparts, and the RT-qPCR results of these specimen showed that chemerin expression was significantly reduced in most (19) of the samples. Furthermore, chi-squared analysis of chemerin expression levels in NSCLC specimen, distinguished between low and high based on a mean score, found that tissue chemerin expression levels were associated with tumor-node metastasis stage, differentiation, and lymph node metastasis (24, 27). Both studies identified tissue chemerin levels as an independent prognostic factor for NSCLC and reported that, overall, higher chemerin expression was associated with positive prognosis (24, 27).

Unlike tissue expression levels, elevated serum chemerin levels were observed in NSCLC patients, which correlated to poorer overall survival, and was identified as an independent risk factor for poorer prognosis in NSCLC patients (97). In a study by Xu et al. (97), analysis of serum samples from 189 NSCLC patients and 120 healthy individuals revealed that serum chemerin levels were upregulated in NSCLC patients. Furthermore, both early stage and advanced stage NSCLC patients showed elevated levels of chemerin in serum, with a further increase for patients with advanced stage NSCLC. For diagnosis of NSCLC, serum chemerin levels had a test sensitivity and specificity of 62.4% and 67.5%, respectively. When combined with carcinoembryonic antigen tests, the sensitivity and specificity of the test increased to 78.3 and 84.2%, respectively. Thus, serum chemerin expression was also established as a viable biomarker for diagnosis and prognosis of NSCLC (97).

To elucidate chemerin's mechanistic roles in lung cancer, Unver et al. (31) examined chemerin's functions in Lewis lung carcinoma (LLC), a mouse model of lung cancer. LLC cells were genetically altered to secrete prochemerin at low levels insufficient to induce chemotaxis. Media from the modified LLC cells was used to culture splenic leukocytes and suppress their expression of inflammatory cytokines, TNF-α, and IL-12 p40 (31). Results were replicated *in vivo*, as expression of TNF-α and IL-12 p40 was also reduced in the prochemerin expressing LLC tumor tissues. These observations are notable because inflammation, though sometimes necessary for antitumor activity, is often exploited by tumors to establish a pro-tumor environment, particularly in cases of NSCLC (98, 99). In syngeneic C57BL6 mice implanted with prochemerin-expressing LLC grafts, tumor formation was impeded by prochemerin expression (31). However, in mice with successful tumor formation, control tumors and prochemerin expressing tumors showed no difference in growth rate, indicating that prochemerin expression may play a role in modulating tumorigenesis through reduced inflammation but not in tumor suppression of LLC (31).

### Prostate cancer

Prostate cancer is one of the most frequently diagnosed cancers in the United States, with more than 164,000 new cases per year (100). It is also the second leading cause of cancer-related mortality in the country (101). Treatment of prostate cancer mainly consists of surgery and radiotherapy for localized cases and androgen deprivation therapy and chemotherapy for metastatic cases (102).

Studies have shown that chemerin is significantly downregulated in prostate cancer. Both analyses of public datasets and RT-qPCR of human prostate tumor samples in several studies demonstrated reduced expression of chemerin, indicating that prostate tumors may downregulate chemerin as a means of immune evasion (15, 26, 103, 104). Thus, it is possible that therapeutic application of chemerin in prostate cancer may enhance anti-tumor immunity and slow tumor progression.

### Squamous cell carcinoma of the esophagus

Esophageal cancer affects millions of people worldwide, with approximately half a million new cases being diagnosed each year (105). Of those cases, esophageal squamous cell carcinoma (ESCC) accounts for a dominant majority, 90% (105). The outlook for patients is bleak, as esophageal cancers are among the deadliest tumors in the world due to its fast progression and late diagnosis (106). Standard therapy for esophageal cancer patients consists of esophagectomy, radiotherapy, and/or chemotherapy (106).

Identifying chemerin's role in ESCC, kumar et al. (20) showed that chemerin was upregulated in ESCC myofibroblasts, which also expressed CMKLR1 (20). Moreover, chemerin was shown to increase invasiveness of ESCC *in vitro*, which was mediated by the accumulation of MMP-1, MMP-2, and MMP-3. In Boyden chambers, chemerin stimulated the migration and invasion of OE21 cells, a human ESCC cell line, and this effect was inhibited by antagonists of CMKLR1 and chemerin siRNA, supporting previous results. Analysis of the OE21 media showed that there was increased expression of MMP-1, MMP-2, and MMP-3, and this effect could be reduced by inhibiting p44/42 MAPK kinase and protein kinase C, showing that they were responsible for increased MMP expression levels (20). Combined with previous reports that MMP-1 expression is associated with poor prognosis in esophageal cancer, the study suggested that chemerin could elicit pro-tumor effects in ESCC (107–109). Thus, it was postulated that targeting chemerin/CMKLR1 may be a viable therapeutic approach for ESCC.

### Squamous cell carcinoma of the oral tongue

Squamous cell carcinoma of the oral tongue (SCCOT) is the most common form of oral squamous cell carcinoma (OSCC), which has a worldwide incidence rate of ~6.6 cases per 100,000 males and 2.9 cases per 100,000 females (110). Current treatment for SCCOT ranges from surgery alone, for stage 1 and 2 cases, to a combination of surgery and radiotherapy for later stages of disease (111). Though early diagnosis and treatment methods are currently available, the primary causes of mortality in SCCOT patients are lymph node metastasis and recurrence (112). Thus, biomarkers for those parameters may be of great prognostic value for SCCOT patients.

A study by Wang et al. (18), looking at chemerin mRNA expression in 19 SCCOT tumor tissues and matched adjacent tissues via qRT-PCR and IHC stains of 147 SCCOT specimen and their associated peritumoral, healthy tissues, determined that both chemerin mRNA and protein was upregulated in primary SCCOT specimens (18). Significantly, overexpression of chemerin was correlated with poor differentiation, high clinical stage, and lymph node metastasis. Multivariate survival analysis further showed that chemerin was an independent prognostic factor for SCCOT and that patients with overexpressed chemerin had a shorter cancer-related survival time (18). Though chemerin's role in SCCOT tumor progression is still unclear, the results indicate that chemerin may be a therapeutic target for inhibiting tumor growth.

Another study found that serum and salivary levels of chemerin were also elevated in OSCC patients, indicating that chemerin may be used to diagnose patients (113). The study included serum and salivary samples from 15 patients with early stage OSCC, 15 patients with oral premalignant lesions (OPML), and 15 healthy individuals, and analysis of these specimen showed that serum and salivary levels of chemerin, and MMP-9, were upregulated in OSCC compared to OPML, and in OPML compared to healthy patients. Analysis of the ROC curve revealed that serum and salivary chemerin levels had a test sensitivity and specificity of 100% for detecting early stage OSCC (113).

### Squamous cell carcinoma of the skin

Cutaneous Squamous Cell Carcinoma (CSCC) is the second most prevalent form of skin cancer in the world (114). Affecting the elderly at disproportionately higher rates, CSCC is mainly caused by chronic exposure to ultraviolet radiation (114, 115). Fortunately, advances in treatment have made new, targeted molecular therapies available to patients, in addition to the standard options of surgery and chemotherapy (116).

A recent study indicated that chemerin may contribute to CSCC cell migration and tumor growth. The study by Farsam et al. (19) found that chemerin was downregulated in CSCC but upregulated in senescent fibroblasts and skin samples of elderly patients (19, 117). Transcriptional analysis of CCRL2 in CSCC cell lines further revealed that CCRL2 expression was significantly increased on CSCC tumor cells *in vitro*, and enhanced tumor cell migration was observed as a result of increased levels of senescence-associated chemerin, which was abrogated by inhibition of chemerin in senescent fibroblasts. Moreover, CMKLR1, or the combination of CCRL2 and GPR1, was necessary for CSCC cell migration via chemerin, given that suppression of either one of the receptors reversed these effects. Finally, chemerin was shown to activate MAPK signaling in SCL-1 cells, a human CSCC cell line, with high expression of CCRL2 and low expression of GPR1. Specifically, the JNK and ERK 1/2 pathways were indicated as the primary mediators of chemerin-mediated effects in CSCC tumor cells, as inhibition of these pathways neutralized the previously observed migratory response. Taken together, these results showed that chemerin enhanced CSCC cell migration and promoted tumor growth through chemerin/CMKLR1/CCRL2/GPR1 interactions and subsequent activation of MAPK pathway subtypes (19).

## Conclusion

Chemerin is a versatile protein with significant functions in modulating tumor growth. With altered expression profiles in a variety of cancer types, it can have context dependent effects on tumorigenesis and tumor progression. In some cases, cancers may silence RARRES2 via hypermethylation to evade immune surveillance. In others, host systems may increase chemerin expression as a defensive measure, recruiting antitumor immunocytes, reducing secretion of inflammatory cytokines, and/or modifying signaling pathways. Through both immune-independent and immune-dependent mechanisms, chemerin has been shown to elicit tumor-suppressive effects in various cancers, and there is a strong possibility that the mechanisms of tumor inhibition seen in these tumor types may be replicated in others as well. Importantly, for cases in which silencing of RARRES2 has been reported, the restoration and/or forced overexpression of chemerin in the microtumor environment may incite compelling antitumor effects, indicating new avenues of research for chemerin in cancer.

Here, we have comprehensively reviewed the current data on chemerin's functions in cancer, along with its underlying mechanisms when available. Concentrating on aspects that may lead to clinical applications, chemerin's diagnostic and/or prognostic value have also been evaluated in a few cancer types. Given the efficacy of chemerin-mediated anti-tumor responses seen now in several tumor settings, future research investigating chemerin's roles in additional tumor types is warranted, with a particular focus on manifesting its therapeutic potential for cancer treatment. Importantly, it should be noted that chemerin-based treatments may add to the efficacy of approved checkpoint inhibitors, as they likely act through independent mechanisms to diminish tumor growth.

## Author contributions

All authors listed have made a substantial, direct and intellectual contribution to the work, and approved it for publication.

### Conflict of interest statement

The authors declare that the research was conducted in the absence of any commercial or financial relationships that could be construed as a potential conflict of interest.
